# Efficiently Visible-Light Driven Photoelectrocatalytic Oxidation of As(III) at Low Positive Biasing Using Pt/TiO_2_ Nanotube Electrode

**DOI:** 10.1186/s11671-016-1248-5

**Published:** 2016-01-19

**Authors:** Yanyan Qin, Yilian Li, Zhen Tian, Yangling Wu, Yanping Cui

**Affiliations:** School of Environmental Studies, China University of Geosciences, Wuhan, 430074 China

**Keywords:** Pt/TiO_2_ nanotubes, As(III), Anode electrolytic tanks, Photoelectrocatalytic oxidation

## Abstract

A constant current deposition method was selected to load highly dispersed Pt nanoparticles on TiO_2_ nanotubes in this paper, to extend the excited spectrum range of TiO_2_-based photocatalysts to visible light. The morphology, elemental composition, and light absorption capability of as-obtained Pt/TiO_2_ nanotubes electrodes were characterized by FE-SEM, energy dispersive spectrometer (EDS), X-ray photoelectron spectrometer (XPS), and UV-vis spectrometer. The photocatalytic and photoelectrocatalytic oxidation of As(III) using a Pt/TiO_2_ nanotube arrays electrode under visible light (*λ* > 420 nm) irradiation were investigated in a divided anode/cathode electrolytic tank. Compared with pure TiO_2_ which had no As(III) oxidation capacity under visible light, Pt/TiO_2_ nanotubes exhibited excellent visible-light photocatalytic performance toward As(III), even at dark condition. In anodic cell, As(III) could be oxidized with high efficiency by photoelectrochemical process with only 1.2 V positive biasing. Experimental results showed that photoelectrocatalytic oxidation process of As(III) could be well described by pseudo-first-order kinetic model. Rate constants depended on initial concentration of As(III), applied bias potential and solution pH. At the same time, it was interesting to find that in cathode cell, As(III) was also continuously oxidized to As(V). Furthermore, high-arsenic groundwater sample (25 m underground) with 0.32 mg/L As(III) and 0.35 mg/L As(V), which was collected from Daying Village, Datong basin, Northern China, could totally transform to As(V) after 200 min under visible light in this system.

## Background

Arsenic (As) contamination is widely recognized as a global health problem. The distribution of As(III) and As(V) in natural water depends on the redox potential and pH of water [1]. Compared with As(V), As(III) is generally reported to have a low affinity to the surface of various minerals, because it mainly exists as nonionic H_3_AsO_3_ in natural water when pH <9. Nevertheless, As(V) adsorbs easily to solid surfaces, so it is easier to be removed. Since As(III) is more toxic and more difficult to remove than As(V), a pre-oxidation technology by transforming As(III) to As(V) is highly desirable to remove arsenic from water [2].

Kinds of treatment methods have been reported on oxidizing As(III) to As(V), including biological oxidation, chemical oxidation with conventional oxidants, such as chlorine, chlorine dioxide (ClO_2_), chloroamine (NH_2_Cl), permanganate (MnO_4_^−^), manganese oxides, and hydrogen peroxide [3], photo-oxidation using ultraviolet and visible light radiation, and photocatalytic oxidation [4]. Among these techniques, the photocatalytic oxidation of As(III) to As(V) is newly developed and becoming a promising method. Up to now, the photocatalysts used for oxidizing As(III) reported in literatures are TiO_2_ [5, 6], BiOI [7], and WO_3_ [8], and TiO_2_ is widely used for As(III) oxidation. Photocatalytic oxidation of As(III) in TiO_2_ suspensions has been proved to be an efficient and environmentally acceptable technique [9–12]. Rapid oxidation from As(III) to As(V) could be realized in TiO_2_ suspensions, e.g., a 10 mg/L of As(III) could be totally oxidized to As(V) within minutes under UV irradiation [13]. TiO_2_ is limited as an efficient photocatalyst because of its wide band gap (3.2 eV) and high recombination rate of photogenerated electron-hole pairs. So, how to expand the absorption band of TiO_2_-based photocatalysts to visible light range or reduce the recombination of electron-hole pairs are key points in using TiO_2_-based materials as highly efficient photocatalysts.

Numerous attempts have been devoted to extend the photo response range of TiO_2_ to visible spectral area. For instance, TiO_2_-based photocatalysts were modified by doping with metal cations [14] or nonmetal ions [15], photosensitizing with dyes on the TiO_2_ surface, depositing noble metals [16], or coupling with another semiconductor (such as CdS, Fe_2_O_3_, ZnO, and SnO_2_) [17, 18]. Up to now, TiO_2_-based nanoparticles functionalized with Fe [19], *γ*-Fe_2_O_3_ [20], Mn_3_O_4_ [21] and MoO_x_ [22]_,_ and sensitized with ruthenium dye [23, 24] have been used for arsenite oxidation and all exhibited better photocatalytic oxidation performance for arsenite than pure TiO_2_. Among these studies, the deposition of Pt nanoparticles on TiO_2_ was proved to have a high photocatalytic activity [25]. Pt doping of TiO_2_ can form the Schottky barrier among the metals and the electronic potential barrier at the metal-semiconductor heterojunction, and the platinized TiO_2_ can trap the photogenerated electrons efficiently [26, 27]. Furthermore, Pt-doped TiO_2_ materials produce significantly higher photocatalytic activity under visible light irradiation, while the photocatalytic activity under UV irradiation is improved slightly.

To reduce the recombination of photogenerated electron-hole pairs, the technique of photoelectrocatalytic oxidation has attracted increasing attention in the field of environmental protection. Photoelectrocatalytic oxidation techniques were first applied in As(III) oxidation under UV irradiation by Fei et al., and the application of an external positive bias voltage on the catalyst could draw the photo-generated electrons away via the external circuit, leaving the holes for oxidation of As(III). Therefore, compared to the photocatalytic process, the probability of the rapid recombination of electron-hole pairs is largely reduced and the photo-oxidation ability for As(III) can be raised in the photoelectrocatalytic process [28]. Later, dye-sensitized photoelectrocatalytic oxidation over nanostructured TiO_2_ film electrodes were applied in As(III) transformation under visible light by Li et al. and showed a high photocatalytic activity for As(III) oxidation [23, 24, 29].

Compared with TiO_2_ film as a photocatalytic electrode, TiO_2_ nanotube arrays fabricated by electrochemical anodization have been demonstrated to be a promising photoanode because of their good physical and chemical properties, large specific surface area, facile synthesis process, and high stability in acidic and alkaline solutions [30, 31]. So, TiO_2_ nanotubes are widely used as photoelectric catalytic electrode instead of TiO_2_ film, and also usually used as novel and stable support for the noble metal catalysts. It has been demonstrated that Pt dopant can also improve the photoelectrochemical performance of TiO_2_ nanotubes under visible light irradiation [32]. Up to now, TiO_2_ nanotubes and Pt/TiO_2_ nanotubes have not been used in photocatalytic oxidation for As(III).

In this paper, constant current deposition [32] method was selected to synthesize Pt/TiO_2_ nanotubes electrode, which was proved to have smaller band gap and stronger absorption in visible light region. These Pt/TiO_2_ nanotubes materials were firstly tried to photoelectrocatalytic oxidation for As(III) in water driven by visible light. The photoelectrochemical oxidation performances of these materials for As(III) separately under visible light and sunlight were tested. The kinetics process of As(III) photoelectrochemical oxidation was analyzed to fit the pseudo-first-order reaction model equation. Real sample from Daying Village, Datong basin, Northern China, with high concentration of As(III) was tried by this system under visible light, and all As(III) was found to be transformed into As(V) in 200 min.

## Methods

### Reagents

All reagents were obtained from Sinopharm Chemical Reagent Co., Ltd. and were the highest grade available. All solutions and subsequent dilutions were prepared using deionized water from a scientific nanopure water purifier (Thermo fisher, America) with a resistivity of less than 0.055 μS/cm. A 1000 mL of As(III) standard solution (1000 mg/L) was prepared by dissolving 1.3203 g of As_2_O_3_ in the minimum amount of 4.0 M NaOH and then adjusting pH to 3.0 with 1.0 M H_2_SO_4_.

### Instruments

The morphology of the samples was studied with the use of a Hitachi SU8010 field emission scanning electron microscope (FE-SEM).

The analysis of the optical properties was performed on a U-4100 UV-vis spectrophotometer (Hitachi, Japan) in the region of 200–800 nm.

X-ray photoelectron spectroscopy (XPS) analysis was carried out to determine the surface properties of the catalysts using a Physical Electronics PHI model 5700 instrument (a RBD upgraded PHI-5000 C ESCA system, PerkinElmer, America), with Al X-ray source operating at 250 W. The takeoff angle of the sample to analyzer was 45°. Survey spectra were collected at pass energy (PE) of 187.85 eV over a binding energy range from 0 to 1300 eV. High binding energy resolution multiplex data for the individual elements were collected at a PE of 29.55 eV. During all XPS experiments, the pressure inside the vacuum system was maintained at 1 × 10^−9^ Pa. Before the above analysis, all samples were dried under vacuum at 80 °C overnight. Binding energies were calibrated by using the containment carbon (C1s = 284.6 eV).

To detect concentration of arsenic, an ELAN DRC II ICP-MS (PerkinElmer, America) equipped with an atomizer and a spray chamber was used. The ICP-MS normal operating parameters were as follows: RF power 1100 W, lens voltage 7.25 V, nebulizer gas flow rate 0.98 L/min, auxiliary gas flow rate 1.2 L/min, and plasma gas flow rate 15.00 L/min. Arsenic species were separated by Series 200 HPLC (PerkinElmer, America) with an automatic sample injector and directly introduced into ICP-MS. A C8 chromatographic column (PerkinElmer, America) was used with the mobile phase containing 1 mM tetrabutylammonium hydroxide, 0.05 mM dipotassium EDTA, and 0.05 % methanol (pH 6.8).

### Preparation of TiO_2_ Nanotubes and Pt/TiO_2_ Nanotubes

**Fig. 1:**
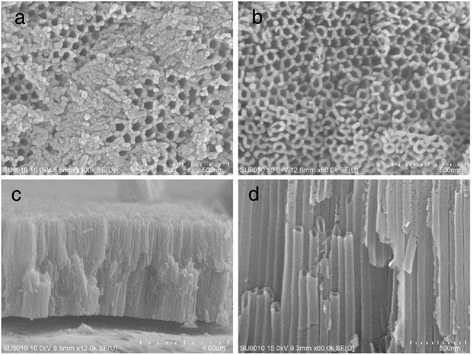
SEM images of annealed TiO_2_ nanotubes: **a** unwashed TiO_2_ nanotubes, **b** washed TiO_2_ nanotubes, **c** low and **d** high magnification of cross-sectional view of washed TiO_2_ nanotubes

Titania nanotubular membranes were fabricated from titanium foil of 0.30 mm thickness (99.9 % pure, Erli, China). Prior to membrane fabrication, the titanium foil was polished with abrasive paper for metallograph, and then ultrasonically cleaned with acetone, ethanol, and de-ionized water, separately for 15 min, and then dried. Then the cleaned titanium foil was set into an electrolyte composed with 0.3 wt.% ammonium fluoride and 2 vol.% water in ethylene glycol. Potentiostatic anodization was done at room temperature with titanium foil (2.0 cm × 3.8 cm) as anode and graphite plate (2.5 cm × 4.5 cm) as cathode. A GPC-6030D constant-voltage DC source (GWinstek, China) was used as the voltage source to drive the anodization. After electrochemical anodic oxidation at voltage of 30.0 V for 2 h, 3.7-μm thick layer of aligned amorphous TiO_2_ nanotubes with 95 ± 5 nm diameters would be presented on Ti sheet. Prepared TiO_2_ nanotubes were ultrasonically cleaned in deionized water for 1–2 min to remove surface debris. Then amorphous TiO_2_ nanotubes layers were converted to the anatase phase by annealing at 450 °C [33]. SEM images of prepared TiO_2_ nanotubes were shown in Fig. 1.

Figure 1a shows the morphology of unwashed TiO_2_ nanotubes after annealing treatment; b–d give the top- and cross-sectional view of washed TiO_2_ nanotubes.

Pt electrodeposition was carried out by using a CS300 electrochemical workstation (Koster, China) with a standard three-electrode system. TiO_2_ nanotubes served as the working electrode, an Ag/AgCl electrode and a graphite plate electrode served as the reference and counter electrode, respectively. Low negative current density (−0.2, −0.3, −0.4, −0.5, −0.6, −0.7, −0.8 mA cm^−2^) with different current-on time was employed to deposit Pt on TiO_2_ nanotubes in the electrolyte. The electrolyte was a mixture of H_2_PtCl_6_·6H_2_O (1.0 g/L), HCl (0.1 mol/L) at 50 °C, pH = 1.0.

**Fig. 2:**
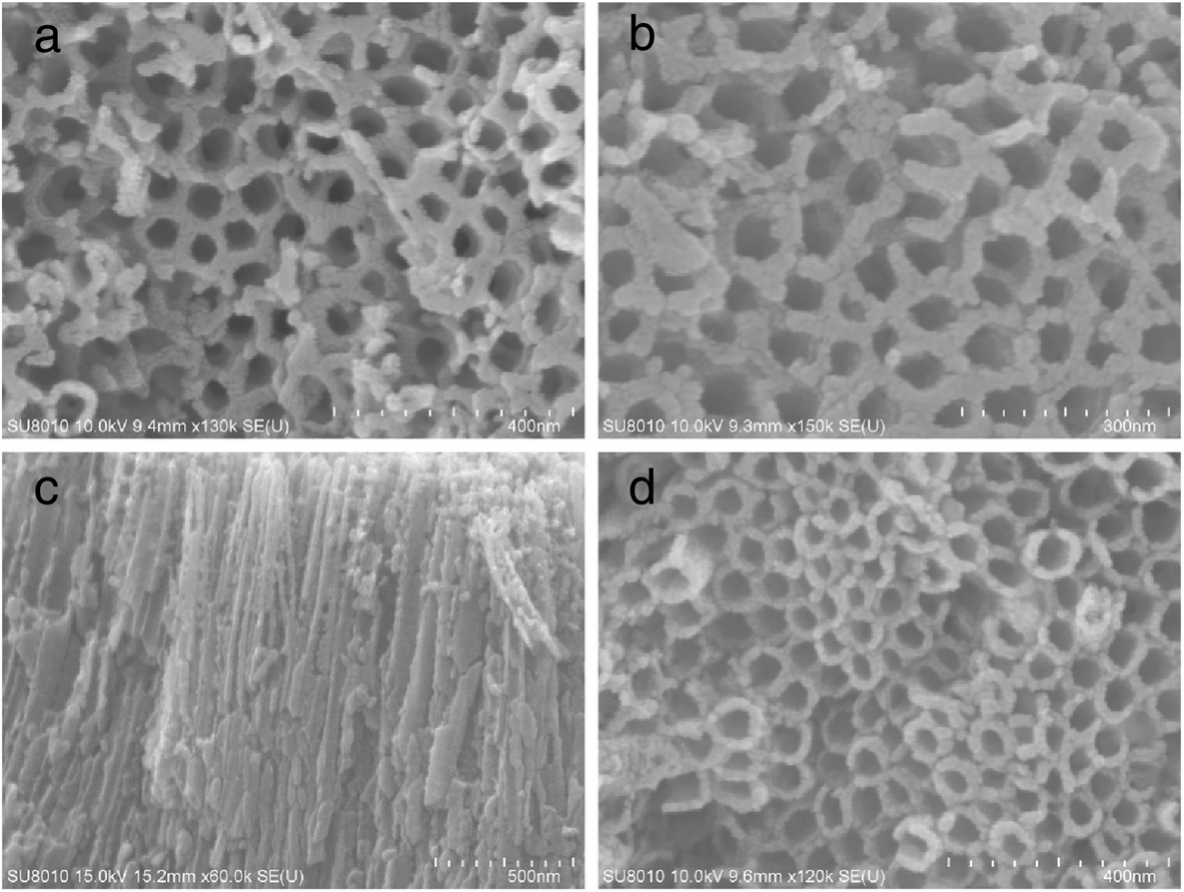
SEM images of Pt/TiO_2_ nanotubes prepared at different current density: **a** 0.2 mA cm^−2^, **b** top view and **c** cross-sectional view with 0.5 mA cm^−2^ of current density, **d** 0.8 mA cm^−2^ current density

Although no obvious Pt particles were observed from the SEM results of Pt/TiO_2_ nanotubes (Fig. 2), energy dispersive spectroscopy (EDS) analysis proved that Pt nanoparticles were existed and focused on the tube wall close to the nozzle (Table 1). Furthermore, from EDS results, the deposition amount of Pt nanoparticles were found steadily increased with the applied current density of Pt deposition.

**Table 1:** EDS results of Pt/TiO_2_ nanotube layers for the whole surface

Element	Current density (mA cm^−2^)							
	0.2 (mA cm^−2^)		0.5 (mA cm^−2^)		0.5 (mA cm^−2^)		0.8 (mA cm^−2^)	
	Top view		Top view		Cross section		Top view	
	Wt%	At%	Wt%	At%	Wt%	At%	Wt%	At%
OK	29.96	58.96	20.12	47.08	40.60	68.85	12.38	35.79
PtM	10.10	1.63	16.10	3.09	5.84	0.81	28.00	6.64
Tik	59.94	39.41	63.78	49.83	53.55	30.33	59.62	57.57

**Fig. 3:**
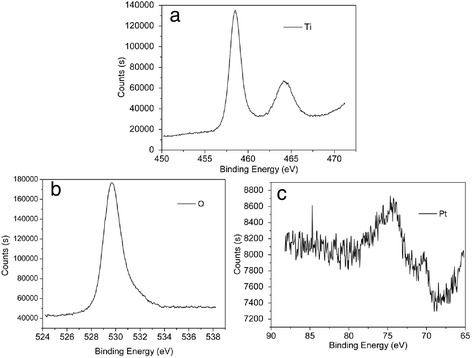
**a** Ti_2p_ peak, **b** O_1s_ peak, and **c** Pt_4f_ peak from XPS spectra of Pt/TiO_2_ nanotubes. The Pt deposition current density was 0.5 mA cm^−2^ and deposition time was 5 min

To further confirm the composition of prepared Pt/TiO_2_ nanotubes, XPS was introduced to detect the surface composition of samples (as shown in Fig. 3). In Fig. 3a, the two peaks at 458.5 and 464.1 eV were assigned to the Ti (2p_3/2_) and Ti (2p_1/2_) states in Pt/TiO_2_ nanotubes, respectively [34]. According to literature, binding energy of Ti^4+^(2p_3/2_) and Ti^3 +^(2p_3/2_) in titanium dioxide was 459 and 457 eV, respectively. The slight peak shift toward low energy suggested that the existence of small amount of Ti^3+^ in the Pt/TiO_2_ nanotubes. The strong peak centered at 529.7 eV corresponded to O(1s) bonded to titanium (Fig. 3b). Compared with the standard O(1s) peak located at 530.0 eV in the XPS spectra of pure TiO_2_ samples, the peak exhibited a 0.3 eV shift to lower energy, which were similar with results reported by Xing et al. [35]. Such shift can be attributed to the lack of oxygen in the Pt/TiO_2_ nanotubes, and oxygen vacancies will be produced with the generation of Ti^3+^ during the preparation process. Ti^3+^ and oxygen vacancies could be generated in the anneal process of TiO_2_ nanotubes and the Pt deposition process, because in the anneal process of TiO_2_ nanotubes oxygen vacancies could not be fully eliminated in current fabrication procedures [36]; at the same time, partial Ti^4+^ would be transformed into Ti^3+^ during the deposition of Pt and Pb with the interaction between Pt/Pb and TiO_2_ [37, 38]. The two peaks located at 70.4 and 74.3 eV shown in Fig. 3c could be assigned to Pt (4f_7/2_) and Pt (4f_5/2_), respectively [39], which indicated that Pt was deposited on the TiO_2_ nanotubes substrate successfully. The binding energy peaks at 70.4 and 74.3 eV are a little higher than that of free Pt nanoparticles (70.3 and 73.6 eV) due to the electrostatic interaction between Pt nanoparticles and TiO_2_ nanotubes [40]. The two Pt_4f_ peaks could be divided into four separated peaks attributed to Pt^0^ (4f_7/2_), Pt^2+^ (4f_7/2_), Pt^0^ (4f_5/2_), and Pt^2+^ (4f_5/2_), and it was found that Pt^0^ was the dominant species in Pt deposited on TiO_2_ nanotubes [41].

**Fig. 4:**
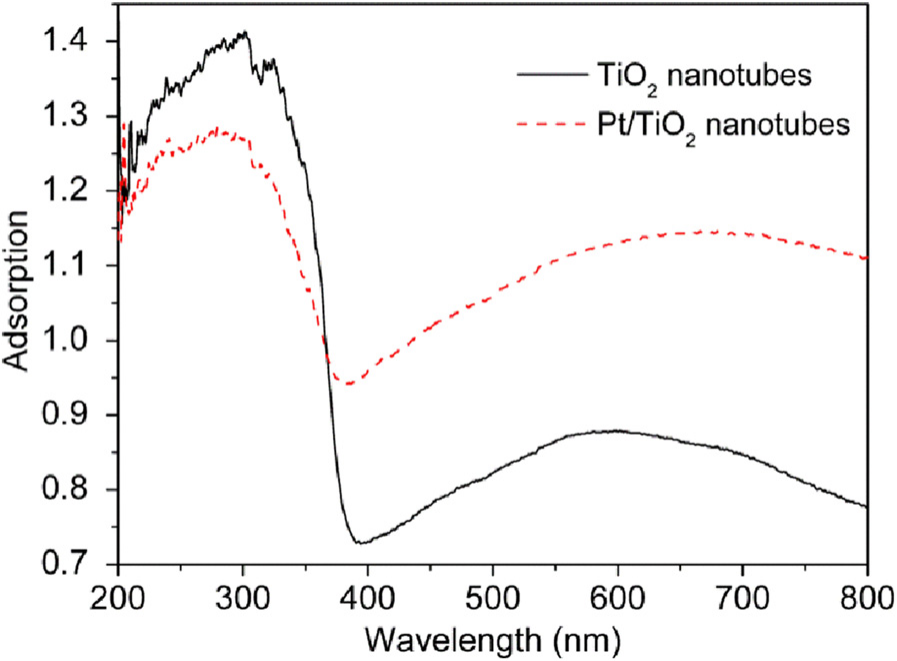
UV-vis diffuse reflectance spectra of TiO_2_ nanotubes and Pt/TiO_2_ nanotubes. The Pt deposition current density was 0.5 mA cm^−2^ and deposition time was 5 min

In order to determine the photo-absorbance properties, the UV-vis diffuse reflectance spectra (DRS) of pure TiO_2_ nanotubes and Pt/TiO_2_ nanotubes was analyzed from 200 to 800 nm wavelengths, as shown in Fig. 4. TiO_2_ nanotubes exhibited a photo-response in ultraviolet region with wavelengths below 390 nm, which could be attributed to intrinsic band gap of TiO_2_. The weak absorption of TiO_2_ nanotubes within the visible light range could be ascribed to the scattering of light caused by pores or cracks in the nanotube arrays or the presence of oxygen vacancies and Ti^3+^ species in the synthesized TiO_2_ nanotubes. Previous researches [36] indicated that in the anneal process of TiO_2_ nanotubes oxygen vacancies and Ti^3+^ species could not be fully eliminated with the current fabrication procedures. Ti^3+^ species could accelerate the formation of isolated defect energy level below the bottom of the conduction band (CB) of TiO_2_, and also absorbed visible light, which would excite and produce photo-generated electrons transforming from Ti^3+^ states to CB of TiO_2_. The weakly visible light absorption of TiO_2_ nanotubes further indicated that oxygen vacancies and Ti^3+^ species probably occurred during the anneal process of TiO_2_ nanotubes. When Pt nanoparticles were loaded on TiO_2_ nanotubes, the photoabsorption amount of the catalyst in visible light region increased and the amount of photoabsorption in the ultraviolet light range decreased. This result was similar to the findings of previous investigations [31]. Compared with pure TiO_2_ nanotubes, the photosensitivity of Pt/TiO_2_ nanotubes in the visible and near visible light wave range increased, because of localized surface plasmon resonance (LSPR) of Pt nanoparticles on the pore-wall of TiO_2_ nanotubes. These results proved, when Pt nanoparticles were loaded on TiO_2_ nanotubes as inorganic sensitizer, the LSPR of Pt nanoparticles promoted the separate efficiency of photogenerated charges and extended the range of the excited spectrum. According to XPS spectrum results, it was demonstrated that oxygen vacancies and Ti^3+^ species were present in Pt/TiO_2_ nanotubes, which induced broad visible light absorption of Pt/TiO_2_ nanotubes. So, Pt/TiO_2_ nanotubes can be tested under visible light to oxidate As(III).

### Photocatalytic Activity Tests

Photocatalytic activities for As(III) oxidation were conducted in a 50-ml quartz beaker. The initial As(III) concentration was fixed at 3.4 mg/L, and the pH was adjusted with H_2_SO_4_ or NaOH solution to the desired value. Prior to As(III) oxidation, TiO_2_ nanotubes was added in the solution and kept for 30 min to allow equilibrium adsorption of arsenite on TiO_2_ nanotubes. UV light irradiation was applied by a 175-W high-pressure mercury lamp, and visible light source was a 300-W halogen lamp (Philips, Holland) equipped with a wavelength cutoff filter for *λ* ≤ 420 nm. Water samples were withdrawn by a 1.0 mL pipette intermittently during photoreaction and filtered through 0.22-μm PTFE filters (Millipore). Duplicate or triplicate experiments were performed for each set.

### Photoelectrocatalytic Activity Tests

**Fig. 5:**
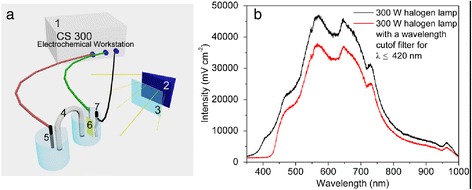
**a** Setup of the photoelectrocatalytic system: *1* CS300 electrochemical workstation, *2* halogen lamp, *3* optical filter, *4* salt bridge, *5* graphite rod, *6* TiO_2_ nanotubes electrode, *7* reference electrode; **b** spectral distribution of the 300-W halogen lamp with and without optical filter

Photoelectrocatalytic oxidation of As(III) was performed in a self-made divided electrolytic tank (Fig. 5a). The anode tank and the cathode tank were isolated, and formed a circuit by a salt bridge. The CS300 electrochemical workstation (Koster, China) was employed to provide constant positive bias voltages, meanwhile, recorded the corresponding current. The Pt/TiO_2_ nanotubes served as working electrodes, with 2.0 × 3.8 cm^2^ area. A saturated calomel electrode and a graphite rod served as reference electrode and auxiliary electrode, respectively. A 50.0 mL electrolyte was comprised of 0.1 M Na_2_SO_4_ (as supporting electrolyte) and As(III) with 2.0, 2.8, 3.4, 4.0, 5.0, and 6.0 mg/L initial concentration. Prior to As(III) oxidation, Pt/TiO_2_ nanotubes working electrode was kept in the electrolyte under darkness for 30 min to ensure adsorption equilibrium. The light source was provided by the 300-W halogen lamp (Philips, Holland) in full wavelength range with illumination intensity around 453 mW cm^−2^ (Fig. 5b). The photocatalytic activity under visible light irradiation (the 300-W halogen lamp) was tested with a cutoff filter to get rid of UV irradiation below 420 nm. To avoid the heating effect caused by the infrared irradiation, the quartz cell was cooled down by circulating water.

## Results and Discussion

### Photocatalytic Oxidation for As(III) by Pt/TiO_2_ Nanotubes Prepared with Different Current Density and Pt Deposition Time

**Fig. 6:**
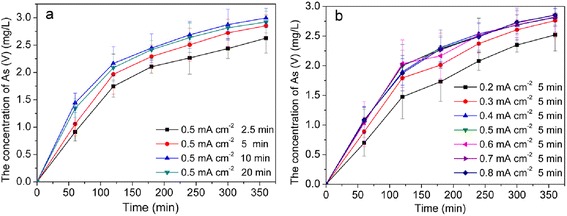
Effect of Pt deposition time (**a**) and Pt deposition current density (**b**) on photocatalytic oxidation for As(III) using Pt/TiO_2_ nanotubes electrodes

The effect of Pt loading time on photocatalytic oxidation As(III) was tested at 25 °C constant temperature in 3.4 mg/L As(III) solution under visible light for 360 min as shown in Fig. 6a. When the Pt loading time were 2.5, 5, 10, and 20 min, the percentages of final dissolved As(V) in system were 77.3, 83.9, 88.1, and 85.9, respectively. Results showed that with the increase of Pt loading time, the oxidation rate of As(III) first increased, then decreased with extensive loading. Considering the economic reason and As(III) oxidation efficiency, the optimal Pt loading time in the following experiments was focused at 5 min.

Figure 6b shows the effect of Pt deposition current density in As(III) photocatalytic oxidation process. When current density of the Pt deposition was increased from 0.2 to 0.8 mA cm^−2^, percentages of final dissolved As(V) were varied from 74.1 to 83.8 %. Concentration of generated As(V) first increased with the increase of applied current density of Pt deposition when it was below 0.4 mA cm^−2^. This is because both the valence state of Pt loaded on Pt/TiO_2_ nanotubes, and the deposition quantity will increase with the applied current density. Photocatalytic activity of platinized TiO_2_ was arranged in the order of Pt (0)/TiO_2_ > PtOx (II, IV)/TiO_2_ > bare TiO_2_ [42]. When applied current density increased to 0.5 mA cm^−2^, the photocatalytic ability of Pt/TiO_2_ nanotubes for As(III) oxidation was kept stable. To the following experiments, the applied current density was kept at 0.5 mA cm^−2^.

### Photocatalytic Ability Comparison Between Naked TiO_2_ Nanotubes and Pt/TiO_2_ Nanotubes

**Fig. 7:**
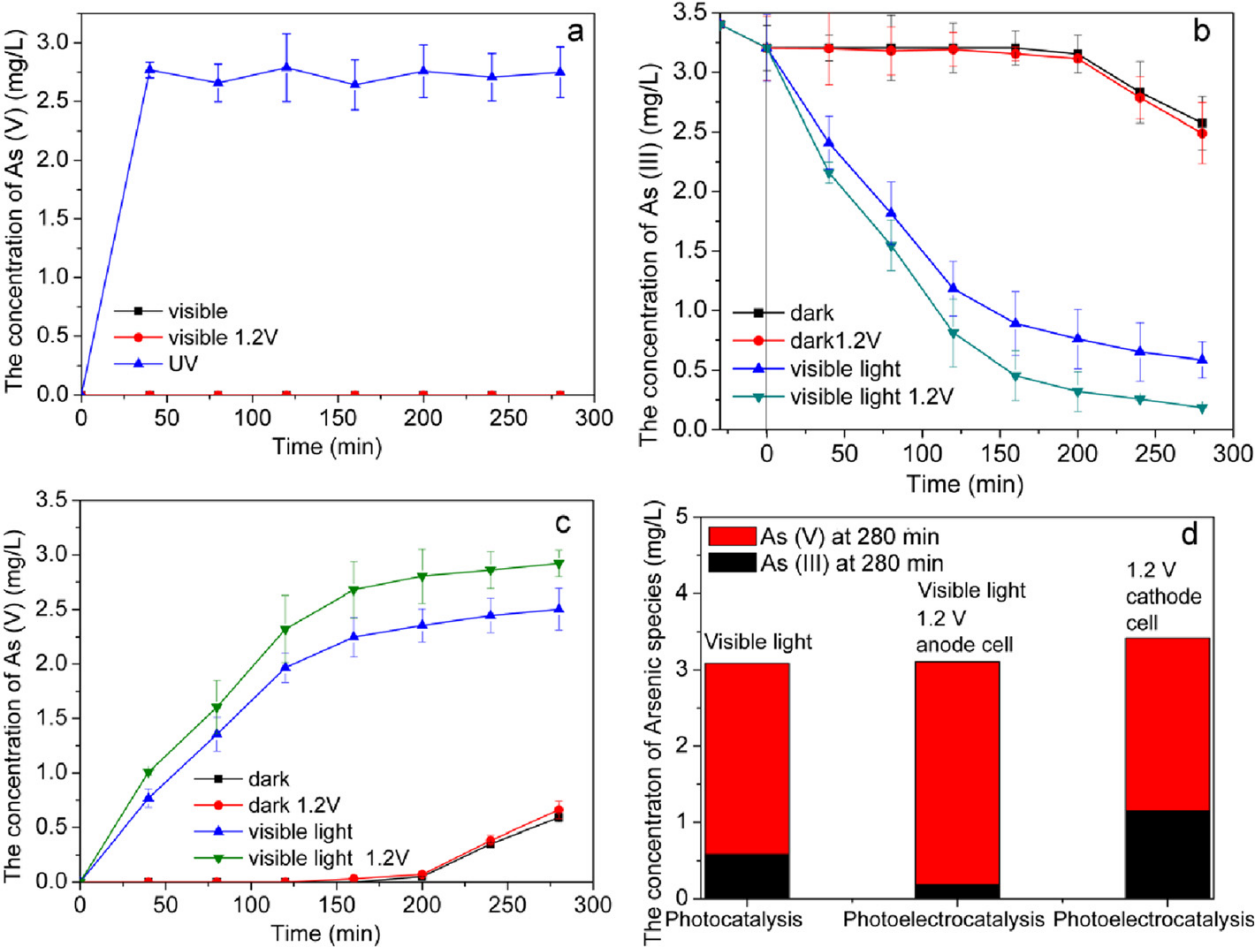
**a** Comparison of As(III) photocatalytic oxidation on TiO_2_ nanotubes under visible light and UV. Comparison of As(III) (**b**) and As(V) (**c**) concentration in system with photocatalytic and photoelectrocatalytic process on Pt/TiO_2_ nanotubes. **d** Concentration of arsenic species in photocatalytic system and photoelectrocatalytic anodic/cathodic cells with Pt/TiO_2_ nanotubes after 280 min

To prove the function of Pt for photocatalysis, oxidation abilities of As(III) were compared between TiO_2_ and Pt/TiO_2_ nanotubes under visible light irradiation or visible light irradiation with 1.2 V positive biasing. From Fig. 7a, we could find that under visible light, no As(III) was oxidized by TiO_2_ nanotubes, no matter if 1.2 V positive biasing was applied. While, under ultraviolet light, 82.0 % of As(III) could be oxidized to As(V) after 30 min. This means, only under ultraviolet light condition, TiO_2_ nanotubes have photocatalytic oxidation ability for As(III).

To Pt/TiO_2_ nanotubes electrodes prepared at 0.5 mA cm^−2^ with 5 min, they displayed high photocatalytic and photoelectrocatalytic oxidation activity for As(III) (Fig. 7 b). To avoid the influence of adsorption effect of Pt/TiO_2_ nanotubes, 30 min equilibrium adsorption was first operated before catalytic experiments, which made As(III) concentration decrease from 3.41 to 3.20 mg/L. When both electrochemical and photocatalytic processes were simultaneously applied, 94.2 % of As(III) could be oxidized in 280 min. This value was 13.5 % higher than the only photocatalytic oxidation process. Fabricated Pt nanoparticles were acted as electron traps, which could enhance the separation of electron-hole pairs, and the external positive biasing drove electron (*e*^−^) to cathode, then the recombination of electron-hole pairs could be further reduced. So, more holes could cause stronger direct (h^+^) oxidation or indirect (HO·) oxidation for As(III) on anode Pt/TiO_2_ nanotubes electrode, which was the reason why the As(III) oxidation rate on anode in the photoelectrocatalytic process was higher than that in the photocatalytic process. In addition, an interesting phenomenon was found on Pt/TiO_2_ nanotubes, As(III) even could be oxidized in dark condition. And 17.4 % of As(III) could be converted into As(V) in 280 min, this could be induced by catalytic effect of platinum itself, and O_2_ activation on Pt nanoparticles might be responsible for this dark activity. When 1.2 V of positive biasing was applied under dark condition, the oxidation efficiency of As(III) to As(V) was hardly improved.

With detection solution in cathode cell, it was interesting to find that As(III) was also continuously transformed into As(V) during the photoelectrocatalytic process at reduction potential. When 1.2 V of positive bias potential was applied to this system, the conversion rate in cathode cell was 66.4 % after 280 min (Fig. 7d).

### Effect of Initial As(III) Concentration on Photoelectrocatalytic Result of Pt/TiO_2_ Nanotubes Electrode

**Fig. 8:**
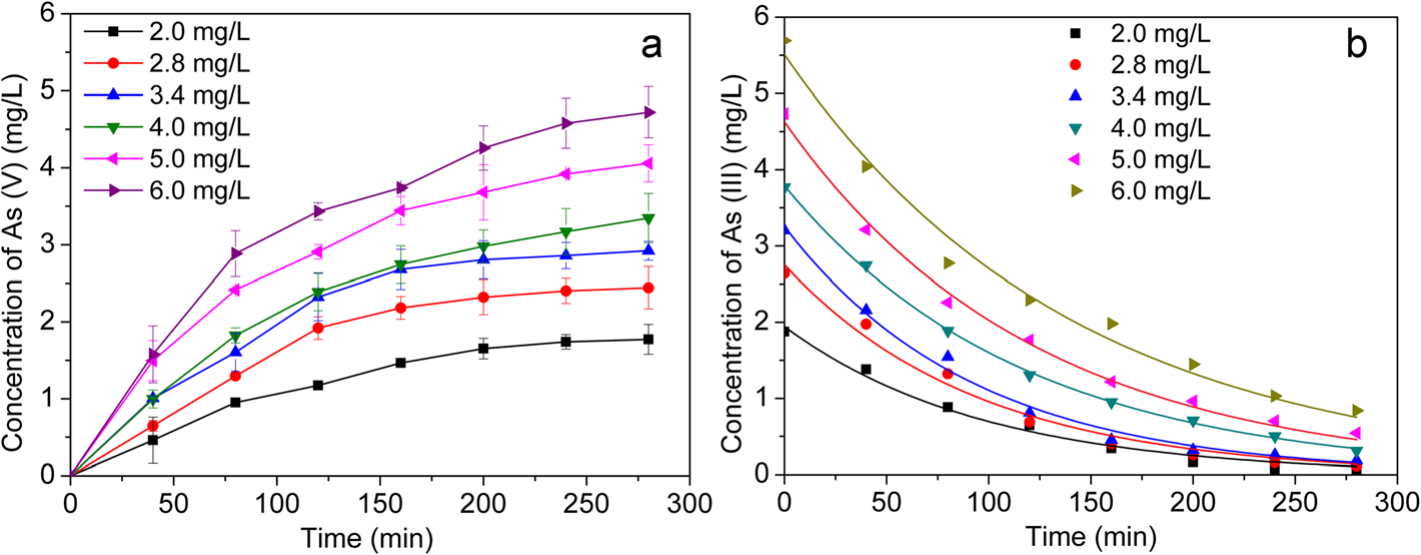
**a** As(V) concentration in anodic cell and **b** kinetics simulation of As(III) oxidation in the photoelectrocatalytic oxidation process with varies initial As(III) concentration

Figure 8a showed the oxidation rate of As(III) increased obviously with the rise of initial As(III) concentration.

Furthermore, the kinetics simulation curves of As(III) photoelectrocatalytic oxidation were summarized and presented in Fig. 8b. All oxidation reactions were well fitted in the pseudo-first-order kinetics model.

$$ - ln\left(\frac{C_t}{C{}_0}\right)={k}_{\mathrm{app}}t $$

where *C* is the concentration of As(III) (mg/L), *t* is the reaction time (min), and *k*_app_ is the apparent first order reaction constant (min^−1^). The values of *k*_app_ and the regression correlation coefficient *R*^2^ of Pt/TiO_2_ nanotubes for As(III) photoelectrocatalytic oxidation were listed in Table 2. Correlation coefficient (*R*^2^) values of pseudo-first-order kinetic model with different initial As(III) concentration were all more than 0.980. This meant that photoelectrocatalytic oxidation process of As(III) on Pt/TiO_2_ nanotubes obeyed pseudo-first-order kinetics equation.

**Table 2:** Kinetic parameters of photoelectrocatalytic oxidation of As(III) with different initial As(III) concentration

Initial As(III) concentration (mg/L)	C_0_(Exp.) (mg/L)	C_0_(cal.) (mg/L)	*R* ^2^	*k*_app_ (min^−1^)
2.0	1.877	1.945	0.984	−0.0103
2.8	2.638	2.753	0.983	−0.0106
3.4	3.204	3.256	0.992	−0.0108
4.0	3.768	3.791	0.999	−0.0086
5.0	4.737	4.630	0.995	−0.0083
6.0	5.692	5.511	0.986	−0.0071

### The Influence of Applied Bias Potentials on the Photoelectrocatalytic Oxidation of As(III)

**Fig. 9:**
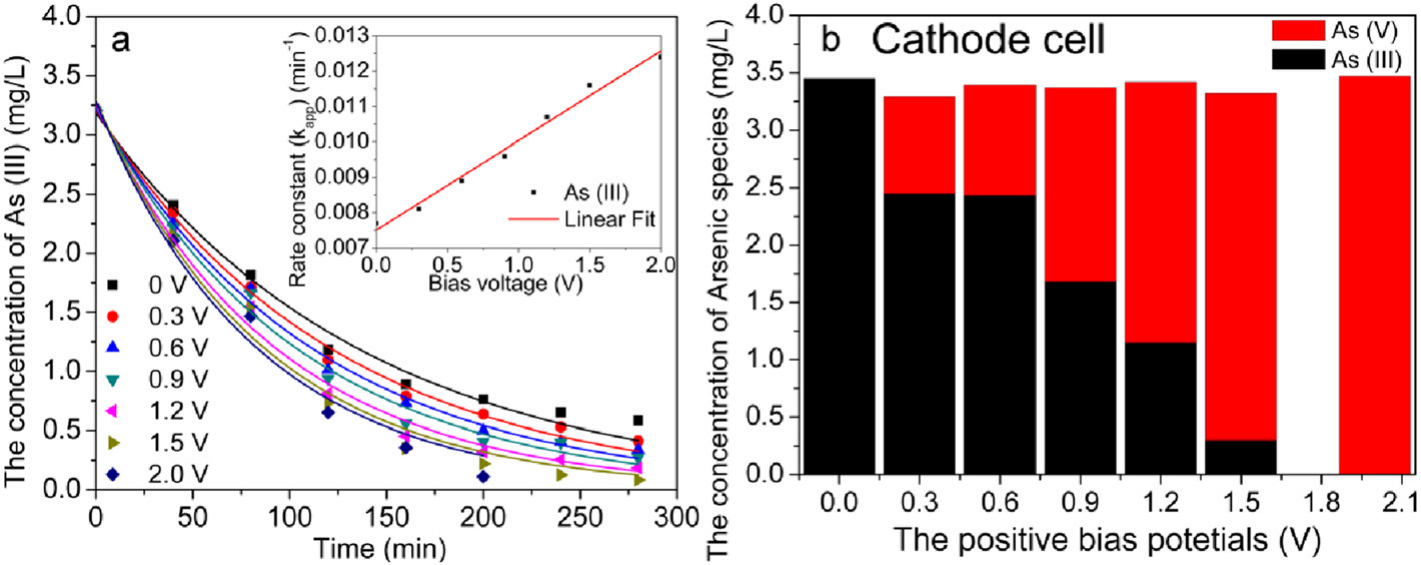
**a** Photocatalytic oxidation kinetics simulation of As(III) at various applied bias potentials, inset was the dependence of rate constant on electrical bias potentials; **b** concentrations of As species in cathode cell with various applied bias potentials after 280 min

The bias potential is an important parameter in the process of photoelectrocatalytic activity. Photo-generated electrons on Pt/TiO_2_ nanotubes electrode could be driven to the counter electrode with positive potential. So, bias potentials ranged from 0.0 to 2.0 V were monitored over 280 min in photoelectrocatalytic oxidation treatment (Fig. 9).

Figure 9a illustrated that As(III) oxidation rate increased with the increase of applied positive bias. With applied 0.0 to 2.0 V positive bias, the plot of *C*_*t*_ versus time was fitted with exponential decay equation with all *R*^2^ exceeding 0.980. The apparent As(III) oxidation rate constants were varied from 7.2 × 10^−3^ to 12.4 × 10^−3^ min^−1^. Applying a positive biasing to the Pt/TiO_2_ nanotubes electrode can transfer the photo-generated electrons away from photo-generated holes on the Pt/TiO_2_ nanotubes electrode via the external circuit; thus, the recombination of photo-generated electron-hole pairs is minimized [43, 44].

In Fig. 9a, it was found that in the range of studied positive biasing, rate constant linearly went up with the increase of applied bias voltage. With the positive bias voltage increasing, more and more photo-generated electrons moved to counter electrode. As a result, the photo-generated electrons and holes were well separated, thus more hydroxyl radicals (HO·) could be produced by H_2_O oxidized in the holes [45]. Which species (h^+^, HO· and ·O_2_^−^) was mainly responsible for the oxidation from As(III) to As(V) in the UV/TiO_2_ system, different opinions had been proposed on this issue. But so far, it still remained as a controversial issue [46]. So, on the anode Pt/TiO_2_ nanotubes electrode, it was in dispute whether h^+^ or HO· was responsible for As(III) oxidation.

At the same time, the oxidation rate of As(III) to As(V) on graphite rod in cathodic cell was also verified to increase with applied potential (Fig. 9b). After 280 min, As(V) concentration were 0, 0.84, 0.96, 1.69, 2.15, 3.02, and 3.39 mg/L when system was applied with 0.0, 0.3, 0.6, 0.9, 1.2, 1.5, and 2.0 V positive bias potentials. As(III) in cathodic cell could be completely transformed into As(V) after 280 min when system was applied with 2.0 V positive bias potential. Leng et al. found that the main product of oxygen reduction reaction was hydrogen peroxide (H_2_O_2_) on the cathode graphite in aqueous solution with pH from 2 to 12 [47]. Nevertheless, ·O_2_^−^ was only stable in concentrated alkaline solutions or aprotic media. So, it was inferred that e^-^ on cathode electrode could be trapped by the surface absorded O_2_ to generate H_2_O_2_ as Eq. (1), which had powerful oxidation ability to convert As(III) to As(V) as Eq. (2) [47].

1 $$ {\mathrm{O}}_2+2{\mathrm{H}}_2\mathrm{O}+2{\mathrm{e}}^{-}\to {\mathrm{H}}_2{\mathrm{O}}_2+2\mathrm{O}{\mathrm{H}}^{-} $$

2 $$ {\mathrm{H}}_3\mathrm{A}\mathrm{s}{\mathrm{O}}_3+{\mathrm{H}}_2{\mathrm{O}}_2+2\mathrm{O}{\mathrm{H}}^{-}\to \mathrm{HAs}{{\mathrm{O}}_4}^{2-}+3{\mathrm{H}}_2\mathrm{O} $$

### The Effect of Solution pH Value on As(III) Oxidation

**Fig. 10:**
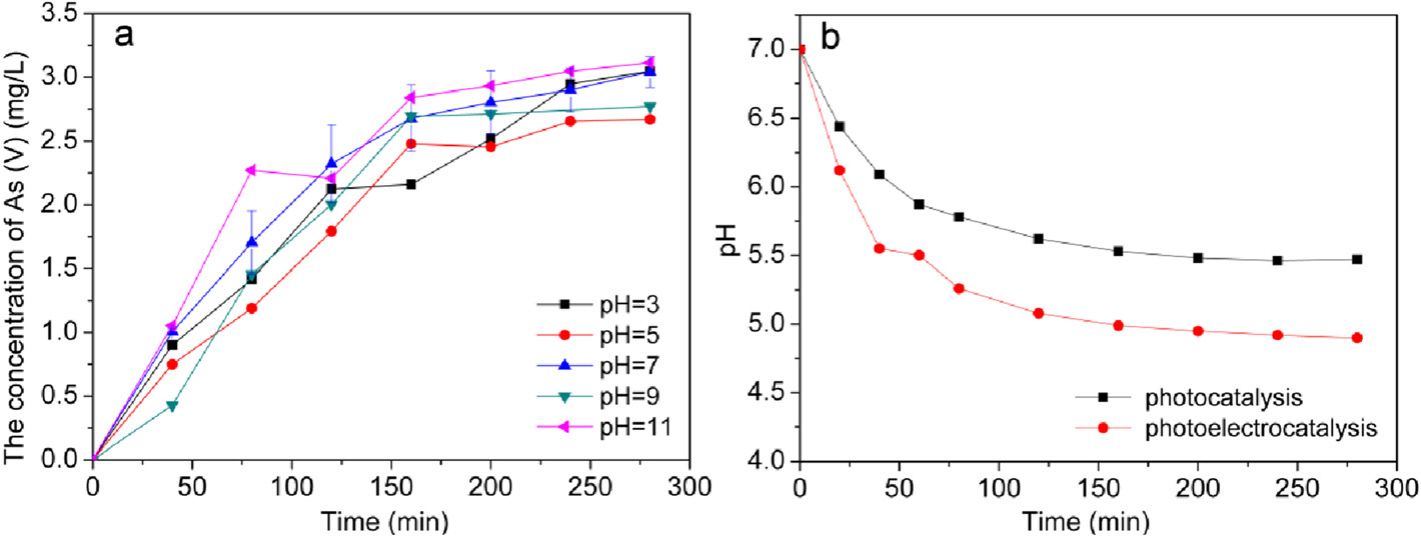
**a** Effect of initial pH on photoelectrocatalytic system; **b** comparison of pH under photocatalytic process and photoelectrocatalytic process (applied 1.2 V) with same initial 7.0 pH and 3.4 mg/L As(III)

The influence of initial pH on photocatalytic oxidation of As(III) in anodic cell was shown in Fig. 10a. The oxidation rate in alkaline environment was mostly a little faster than the rate in acid environment. The result differed from that of Lee and Choi [48] who found that the initial oxidation rate at pH 9 was about twice as fast as the rate at pH 3, and also differed with the result of Bissen et al. [13] and Sharma et al. [49] who both found that the small increase in the oxidation of As(III) with increasing pH was within experimental error.

The potential of As(V)/As(III) couple is much less positive than the valence band potential of TiO_2_, so, the photo-generated holes have enough thermodynamic potential to oxidize As(III) to As(V) [50]. The potential of As(V)/As(III) couple in alkaline environment is lower than that in acid, which may be contributed to the increase of the oxidation rate with the increase of pH.

The pH change during the photoelectrocatalytic oxidation of As(III) in aqueous solution was displayed in Fig. 10b. During the electrochemical experiment, pH decreased with irradiation time during both photocatalysis and photoelectrocatalysis experiments. The decrease of pH in the photoelectrocatalysis experiment was faster than that in the photocatalysis.

The influence of pH on the speciation of arsenic oxoanions can easily be obtained from Eq. (3) to (8), acid dissociation constants are also given.

3 $$ {\mathrm{H}}_3\mathrm{A}\mathrm{s}{\mathrm{O}}_3\leftrightarrow\ {\mathrm{H}}_2\mathrm{A}\mathrm{s}{{\mathrm{O}}_3}^{-} + {\mathrm{H}}^{+}\mathrm{p}{\mathrm{K}}_{\mathrm{a}1}=9.20 $$

4 $$ {\mathrm{H}}_2\mathrm{A}\mathrm{s}{{\mathrm{O}}_3}^{-}\leftrightarrow \mathrm{HAs}{{\mathrm{O}}_3}^{2-}+{\mathrm{H}}^{+}\mathrm{p}{\mathrm{K}}_{\mathrm{a}2}=12.10 $$

5 $$ \mathrm{HAs}{{\mathrm{O}}_3}^{2-}\leftrightarrow \mathrm{A}\mathrm{s}{{\mathrm{O}}_3}^{3-}+{\mathrm{H}}^{+}\mathrm{p}{\mathrm{K}}_{\mathrm{a}3}=13.40 $$

6 $$ {\mathrm{H}}_3\mathrm{A}\mathrm{s}{\mathrm{O}}_4\leftrightarrow {\mathrm{H}}_2\mathrm{A}\mathrm{s}{{\mathrm{O}}_4}^{-}+{\mathrm{H}}^{+}\mathrm{p}{\mathrm{K}}_{\mathrm{a}1}=2.20 $$

7 $$ {\mathrm{H}}_2\mathrm{A}\mathrm{s}{{\mathrm{O}}_4}^{-}\leftrightarrow \mathrm{HAs}{{\mathrm{O}}_4}^{2-}+{\mathrm{H}}^{+}\mathrm{p}{\mathrm{K}}_{\mathrm{a}2}=6.94 $$

8 $$ \mathrm{HAs}{{\mathrm{O}}_4}^{2-}\leftrightarrow \mathrm{A}\mathrm{s}{{\mathrm{O}}_4}^{3-}+{\mathrm{H}}^{+}\mathrm{p}{\mathrm{K}}_{\mathrm{a}3}=11.50 $$

In the photocatalytic process, As(III) was oxidized to As(V), meanwhile, protons were produced, and the reaction could be described as

9 $$ {\mathrm{H}}_3\mathrm{A}\mathrm{s}{\mathrm{O}}_3+{\mathrm{H}}_2\mathrm{O}\ \to\ \mathrm{HAs}{{\mathrm{O}}_4}^{2-} + 4{\mathrm{H}}^{+}+2{e}^{-}\left(\mathrm{in}\ \mathrm{alkaline}\ \mathrm{s}\mathrm{olutions}\right) $$

10 $$ {\mathrm{H}}_3\mathrm{A}\mathrm{s}{\mathrm{O}}_3 + {\mathrm{H}}_2\mathrm{O}\ \to\ {\mathrm{H}}_2\mathrm{A}\mathrm{s}{{\mathrm{O}}_4}^{-} + 3{\mathrm{H}}^{+} + 2{e}^{-}\left(\mathrm{in}\ \mathrm{acidic}\ \mathrm{s}\mathrm{olutions}\right) $$

In photoelectrocatalytic process, the anode oxidation caused O_2_ evolution in anode cell, which produced protons and contributed to the further reduce of the pH during photoelectrocatalytic activity.

### Photoelectrocatalytic Oxidation of As(III) by Natural Sunlight

**Fig. 11:**
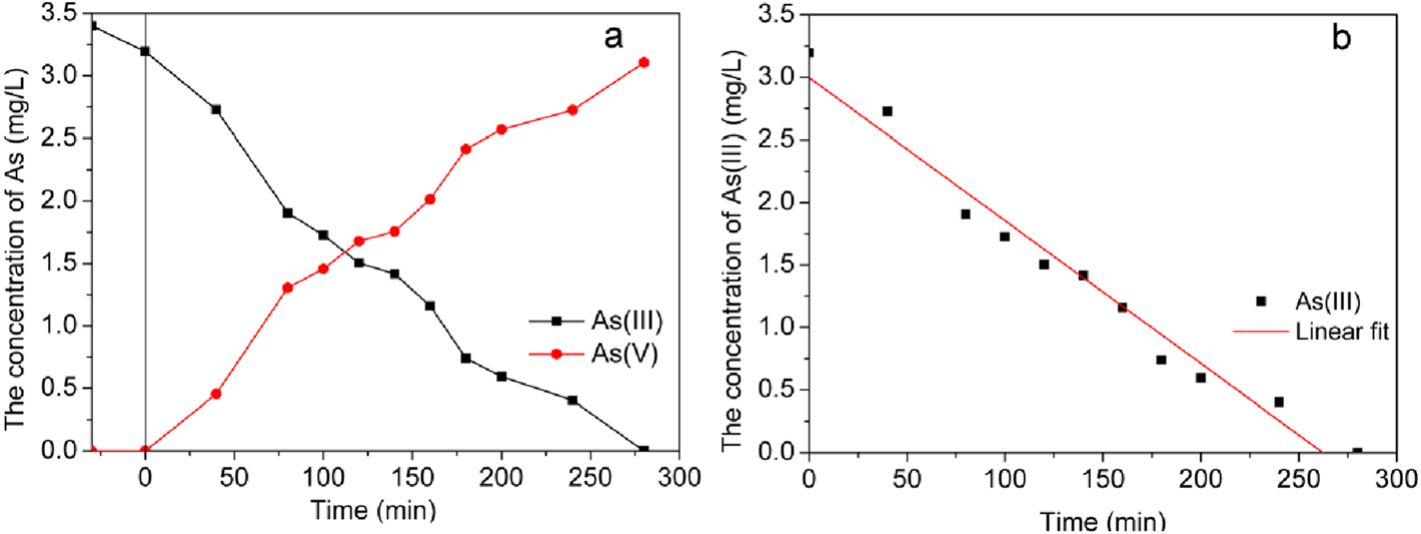
**a** Concentration of As species and **b** kinetics simulation of As(III) oxidation in the process of photoelectrocatalytic oxidation under natural sunlight. Nov. 3rd, 2014, from 10:00 to 14:40, temperature is 16 °C

To further extend the photoelectrocatalytic applicability of Pt/TiO_2_ nanotube arrays electrode in a more practical situation, the photoelectrocatalytic oxidation ability was also evaluated under natural sunlight. Figure 11a showed the evolution of As(III)/As(V) concentration with irradiation time with initial 3.4 mg/L As(III) and 1.2 V positive bias potential under natural sunlight irradiation. The photoelectrocatalytic oxidation process under natural sunlight accorded with zero-order kinetics law (*C*_*t*_ = − 0.0114*t* + 2.9971(*R*^2^ = 0.973)), different with process under visible light which followed first-order kinetics law. As(III) could be completely oxidized after 280 min under natural sunlight irradiation. While, to visible light, it needed 360 min to totally oxidize As(III). This might be because of the effect of ultraviolet light in sunlight.

### Photoelectrocatalytic Oxidation of As(III) in High Arsenic Groundwater Sample Under Visible Light

**Fig. 12:**
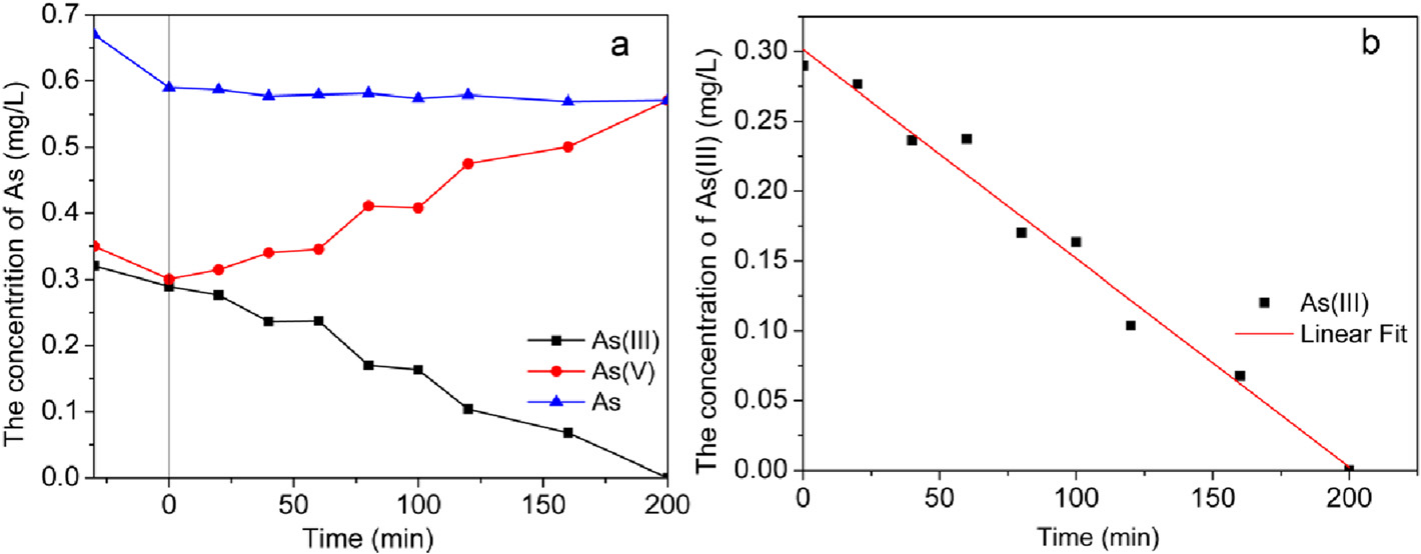
**a** Concentrations of As species and **b** kinetics simulation during the photoelectrocatalytic process with high arsenic groundwater sample under visible light

The photoelectrocatalytic activity of real groundwater sample with high arsenic was also investigated by Pt/TiO_2_ nanotubes arrays electrode under visible light. The high arsenic underground water sample (25 m underground) was collected from Daying Village, Datong basin, Northern China, concentrations of main ions in this water sample was determined as follows: Na^+^ 324.2 mg/L, Ca^2+^ 35.8 mg/L, Mg^2+^ 61.5 mg/L, Cl^−^ 297.4 mg/L, HCO_3_^−^ 544.1 mg/L, SO_4_^2−^ 275.5 mg/L, NO_2_^−^ 1.10 mg/L, Fe^2+^ 0.75 mg/L, NH_4_^+^ 0.81 mg/L, S^2−^ 74.9 μg/L, As(III) 0.32 mg/L, As(V) 0.35 mg/L, 8.32 of pH, and 2.3 mS cm^−2^ of conductivity. After 30 min for equilibrium adsorption, 9.5 % of As(III) and 14.2 % of As(V) were adsorbed on Pt/TiO_2_ nanotube arrays. The conversion of As(III) during the photoelectrocatalytic process with 1.2 V of positive bias potential was found to follow a zero-order kinetics law as *C*_*t*_ = − 0.0015*t* + 0.3013  (*R*^2^ = 0.981), and As(III) in groundwater sample was totally transformed into As(V) in 200 min under visible light. This proved Pt/TiO_2_ nanotubes electrode was an efficient photoelectrocatalytic material for As(III) oxidation and could be used in high arsenic underground water pretreatment (Fig. 12).

## Conclusions

To promote photocatalytic oxidation of As(III) by TiO_2_ materials under visible light, Pt/TiO_2_ nanotubes were introduced via a constant current deposition method. First, the prepared nanotubes were detected by SEM, XPS to prove the existing of Pt, and UV-vis diffuse reflectance spectra results proved that Pt could promote separate efficiency and extend the excited spectrum range. Pt/TiO_2_ nanotubes presented more efficient photoelectrocatalytic oxidation performance for As(III) than TiO_2_ nanotubes. Even under dark condition, it was also useful in As(III) photoelectrocatalytic oxidation. Furthermore, the photoelectrocatalytic oxidation process under visible light was found to obey pseudo-first-order kinetics. The prominent conversion from As(III) to As(V) in cathodic cell also occurred because of the production of H_2_O_2_ from electrons trapping by O_2_ on cathode. While to natural sunlight, the oxidation of 3.4 mg/L As(III) on Pt/TiO_2_ nanotubes electrode with 1.2 V applied voltage followed zero-order kinetics law, and its oxidation rate was slightly higher than that of As(III) under visible light. As(III) in real groundwater sample could be totally transformed into As(V) in 200 min by Pt/TiO_2_ nanotubes electrode with 1.2 V under visible light and also accorded with zero-order kinetics.
